# Navigating the complex terrain of motivated behavior: a bibliometric and neuroscientific perspective

**DOI:** 10.3389/fnbeh.2024.1363856

**Published:** 2024-04-26

**Authors:** Ammir Y. Helou, Jackson C. Bittencourt

**Affiliations:** ^1^Laboratory of Chemical Neuroanatomy, Department of Anatomy, Institute of Biomedical Sciences, Universidade de São Paulo, São Paulo, Brazil; ^2^Center for Neuroscience and Behavior, Institute of Psychology, Universidade de São Paulo, São Paulo, Brazil

**Keywords:** motivation, LDA, neuroscience, reward-seeking, addiction, goal-oriented

## Abstract

Over several decades, motivated behavior has emerged as a crucial study area within neuroscience. Understanding the neural substrates and mechanisms driving behaviors related to reward, addiction, and other motivation forms is pivotal for novel therapeutic interventions. This review provides a bibliometric analysis of the literature, highlighting the main trends, influential authors, and the potential future direction of the field. Utilizing a dataset comprised by 3,150 publications from the Web of Science and Scopus databases (“motivated behavior as query), we delve into key metrics like publication trends, keyword prevalence, author collaborations, citation impacts, and employed an unsupervised natural language processing technique – Latent Dirichlet Allocation – for topic modeling. From early investigations focusing on basic neural mechanism and behaviors in animal models to more recent studies exploring the complex interplay of neurobiological, psychological, and social factors in humans, the field had undergone a remarkable transformation. The last century has seen a proliferation of research dedicated to uncovering the intricacies of motivation, significantly enriching our understanding of its myriad implications for human behavior and mental health. This bibliometric analysis aims to offer comprehensive insights into this dynamic research area, highlighting the field’s key contributions and potential future directions, thereby serving as a valuable resource for researchers, and hopefully give a more thorough understanding of the research area.

## 1 Introduction

The study of what drives us – our motivations – is a fascinating chapter in the story of neuroscience and psychology. It’s a journey that began with a deep dive into the cellular nuts and bolts of our brains and has since woven its way through the rich tapestry of our minds and societies. Think back to the early days when researchers were piecing together the puzzle of the mesolimbic dopamine pathway, uncovering how our brains tally up rewards. This was just the starting point. As time went on, scholars layered in the wisdom of cognitive theories, like the expectancy-value models that gauge the likely payoffs of what we do, all while keeping an eye on how our social worlds and the places we inhabit tug on the strings of our motivations. As understood in modern psychology and neuroscience, motivation has evolved over centuries, with roots in philosophical inquiry, later refined by psychological theories and neuroscientific research (Abdullah, 2019; Wasserman and Wasserman, 2020; Williams, 2023). From the philosophical discussions of Aristotle on ‘telos’ (Greek, the end ‘goal’, ‘purpose’) to the emergence of motivation in psychological terms in the 19th and early 20th centuries, the journey of understanding motivation has been extensive. It encompasses various perspectives, including behaviorism, drive theory, humanistic and cognitive perspectives, and a significant leap in the latter half of the 20th century into the 21st, with the neuroscientific integration of these concepts (Abdullah, 2019; Hattie et al., 2020; Wasserman and Wasserman, 2020; Williams, 2023). The etiology and scientific explanation of the term “motivation” and “motivated behavior” in the context of neuroscience and psychology are rooted in the interplay between physiological needs, psychological states, and environmental stimuli that drive organisms toward goal-oriented actions (Koenka, 2020). “Motivation” derives from the Latin word *movere*, meaning “to move.” The concept has evolved to encompass the reasons or drives behind an organism’s action (Hattie et al., 2020). Motivated behavior has intrigued neuroscientists for many years. The intricate interplay between neural circuits, neurotransmitters, and external stimuli offers a complex field of study.

The study of motivation encompasses a multifaceted approach that integrates biological, neurobiological, cognitive, psychosocial, and evolutionary perspectives. Early instinct theories proposed that behaviors are innate and crucial for survival, whereas the concept of homeostasis and drive highlights the role of internal physiological balance in motivating behavior (Finlay et al., 2011; Gardner, 2011; Sokolowski and Corbin, 2012; Baldo et al., 2013; Alonso-Alonso et al., 2015). At the neurobiological level, the mesolimbic dopamine system, including the ventral tegmental area (VTA) and nucleus accumbens (NAc), plays a central role, with dopamine and other neurotransmitters like serotonin and norepinephrine influencing various aspects of motivated behavior (Ikemoto and Panksepp, 1999; Kelley and Berridge, 2002; Gardner, 2011; Lammel et al., 2012; Sokolowski and Corbin, 2012). Cognitive perspectives, such as expectancy-value models and goal-setting theory, suggest that motivation results from the expectation of achieving desirable outcomes and the value assigned to them, influenced by specific and challenging goals (Hattie et al., 2020; Wasserman and Wasserman, 2020). Psychosocial aspects consider the dichotomy of intrinsic and extrinsic motivation, with Self-Determination Theory emphasizing the need for autonomy, competence, and relatedness for psychological well-being (Deci and Ryan, 2000). Evolutionarily, motivation is seen as an adaptive mechanism promoting survival and reproductive success (Finlay et al., 2011). Clinically, understanding motivation is essential, as alterations in motivated behavior are indicative of psychiatric conditions such as addictive disorders and depression, where reward system dysregulation and motivational deficits are common (Kelley and Berridge, 2002; Gardner, 2011; Sokolowski and Corbin, 2012; Baldo et al., 2013; Williams, 2023). However, an often-overlooked aspect in the study of motivation is apathy, a condition characterized by diminished motivation not attributable to diminished level of consciousness, cognitive impairment, or emotional distress. Apathy presents a significant clinical challenge, particularly in neurological and psychiatric disorders, affecting patient outcomes and quality of life. Its inclusion in discussions of motivated behavior is crucial, as highlighted by seminal works such as Levy and Dubois (2006) and Le Heron et al. (2018), which explore apathy’s implications across neurodegenerative, inflammatory, vascular, and traumatic etiologies.

In the study of motivated behavior, social scientists (like anthropologists, sociologists, and some psychologists) and neuroscientists (here referred to as biologists and biomedical scientists) offer distinctly different perspectives. Social scientists focus on sociocultural, environmental, and psychological factors, emphasizing the complexity of human experience and the influences of external contexts. They often critique biological reductionism for oversimplifying human behavior. In contrast, neuroscientists prioritize understanding the biological perspective of motivation, investigating neural, genetic, and physiological aspects through empirical methods. This naturalistic approach values the objectivity of biological explanations but is sometimes viewed with skepticism in the humanities due to concerns over determinism and the historical misuse of biological explanations (Brigandt, 2013; Bondebjerg, 2015). The main anthropocentric theories of motivation in the 20th century fall into four categories: content, process, cognitive, and humanistic (Berridge, 2018). Content theories, like Maslow’s hierarchy of needs, Herzberg’s two-factor theory, and McClelland’s acquired needs theory, focus on internal factors such as needs, desires, and goals driving human behavior. Process theories, including Skinner’s reinforcement theory, Bandura’s self-efficacy theory, Vroom’s expectancy theory, Locke’s goal-setting theory, and Adams’ equity theory emphasize cognitive processes in how people select and pursue goals. Cognitive theories, like Atkinson’s achievement motivation theory, Deci and Ryan’s self-determination theory, and Weiner’s attribution theory, explore mental processes underpinning motivation. Humanistic theories, represented by Rogers’ person-centered theory and Maslow’s self-transcendence theory, highlight personal growth, self-awareness, and free will in motivation (Berridge, 2018).

Bibliometrics analysis is the quantitative analysis of publications, which provides social and bibliographic insights into scholarly records. This method, primarily ahistorical, can be applied in various contexts to study the development of specific topics over time (Gupta and Bhattacharya, 2004). The growth and development of disciplines are heavily based on scholarly communication, with fields evolving in diverse cultural and intellectual sectors, and the journals serve as a means of communication for academics and researchers, who form communities based on shared disciplinary principles (Gupta and Bhattacharya, 2004). Bibliometrics identifies publication patterns by analyzing the resources used and the intellectual content of published material. These tools, initially utilized by specialists before the 1990s, have become more accessible with the introduction of the Web of Science™ (WoS), Scopus, PubMed, and many other bibliometric databases, thereby making indexed documents available in an online format (Szomszor et al., 2021).

Through bibliometric analysis of the available literature, we aim to distill key trends, notable authors, and foundational papers that have significantly influenced the field. Motivated behaviors are fundamental drivers of human and animal actions, steering organisms toward rewards and away from potential threats. This complex spectrum of behaviors spans from the neurobiological substrates, such as neural circuitry and reward processing, which form the bedrock of motivation, to the myriad expressions of these drives in behaviors like feeding, sexual activity, and the management of addiction. Over the past decades, increasing research has been dedicated to exploring these behaviors, leading to an intricate, multidimensional landscape of interconnected studies. The present work aims to provide a comprehensive bibliometric analysis of this research landscape, offering insights into the field progression, key contributors, major research themes, and emerging trends in motivated behavior research.

## 2 Methodological overview – bibliometric analysis results

Our bibliometric analysis and visualizations of motivated behavior in neuroscience were based on a compiled dataset sourced from both Web of Science™ Core Collection and the Scopus database (data span 1940 to November 2023). From the Web of Science™ Core Collection, querying (“Motivated Behavior”) across all fields yielded 1,485 publications. In the Scopus database, a more focused query, limited to Title, Keywords, and Abstract, retrieved 2,669 papers on the same topic. All field searches found 20,943 documents, though most of them were indirectly related to motivated behavior – the usage of specific terms in the title often reflects a focused study on that concept, as opposed to ‘topic’ searches that can return a wide net of loosely related works (Leng and Leng, 2021). All bibliographic data were downloaded. After merging these datasets, we meticulously excluded documents that contained missing data and duplicates (based on title, from a total of 4,154 documents, 1,001 were duplicates), resulting in a comprehensive collection of 3,153 publications, which 1,150 contain “human” in the index keywords. The number of citations kept were from the highest value found between both datasets, which came from Scopus more frequently than from WoS. This refined dataset formed the basis of our detailed bibliometric analysis (see Supplementary material). All bibliometric data harvested are available in the Supplementary material. The following analysis were performed:

### 2.1 Citation analysis

**Figure 1:**
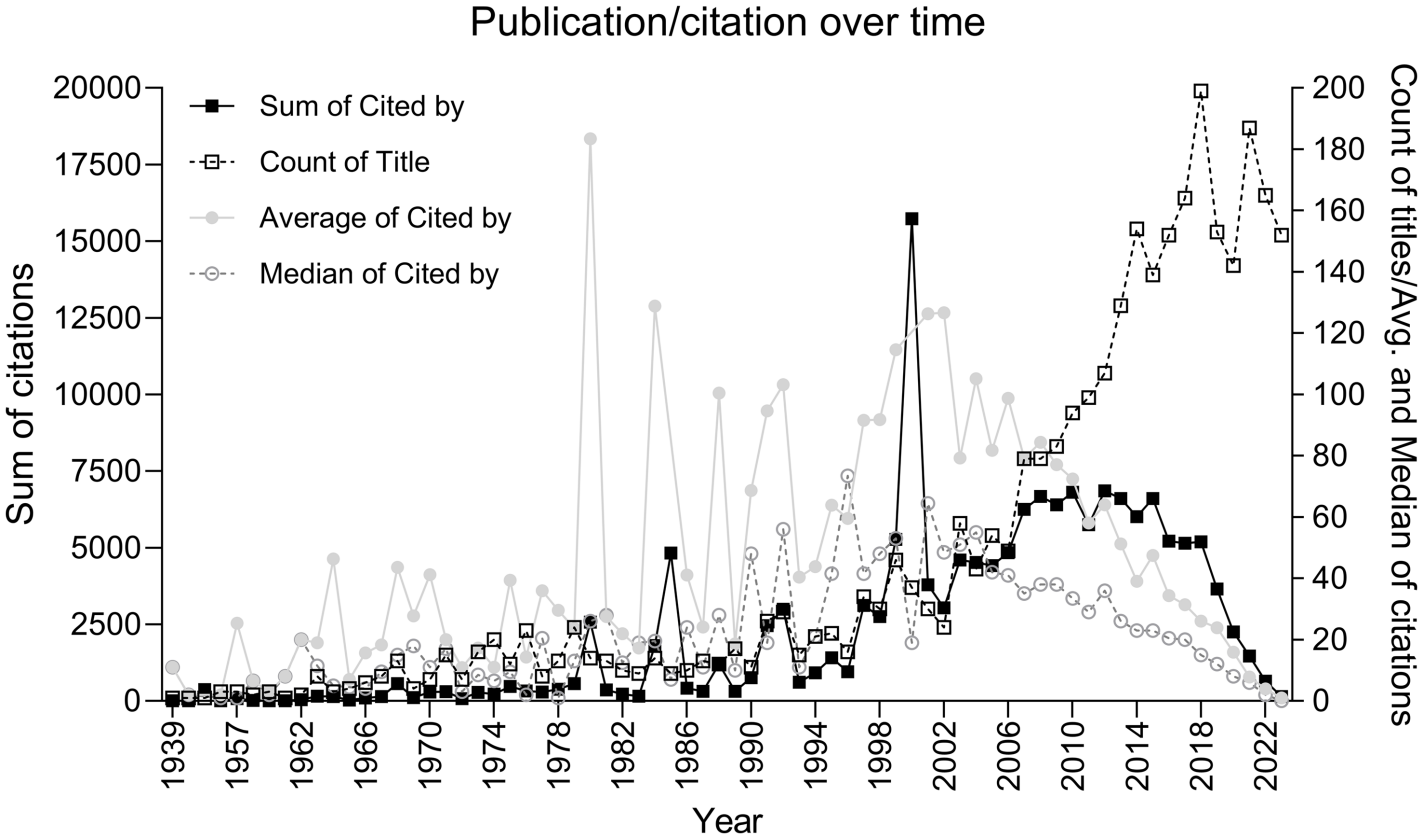
Number of publications and citations overtime per year: count of publications and average, median, and sum of citations over time. The graph elucidates key moments in the field’s history, marked by seminal publications that have garnered considerable attention and have been widely cited within the academic community.

**Figure 2:**
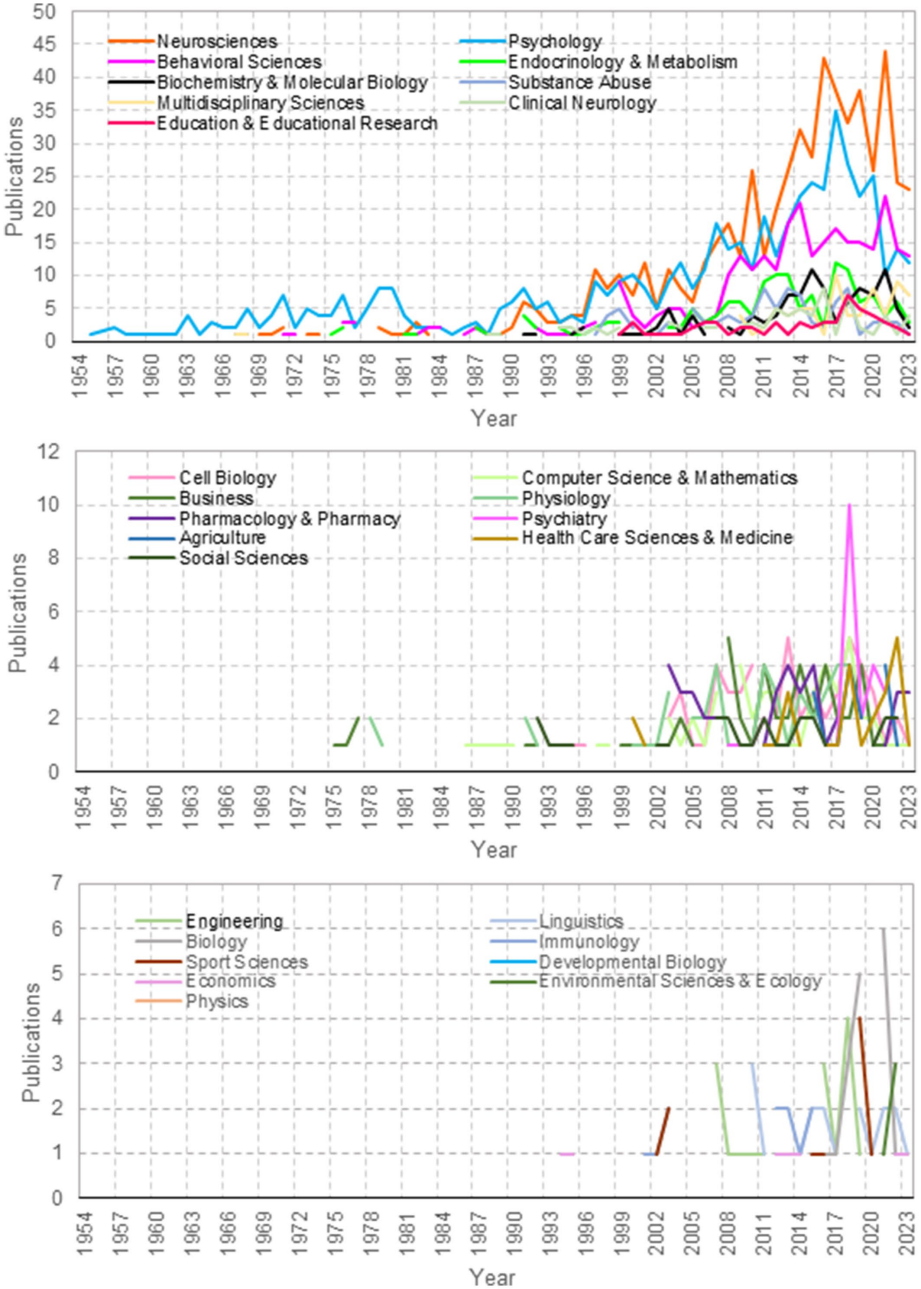
Number of publications per research area over time: number of publications within each research area over time. The lines of varying colors represent distinct research areas. Peaks in the graph not only signify prolific periods of publication but also hint at underlying events or breakthroughs that may have catalyzed these research endeavors. Classification reflects the source databases’ standardized subject categorizations, which may not fully represent all research on motivated behaviors, such as clinical neurology studies on apathy, due to their broader or more specialized indexing criteria. The prominence of fields like substance abuse and addiction is due to their explicit linkage with motivated behavior in the literature. Data span: 1954 to 2023.

Number of total citations per publication was used to calculate the average, median and sum of citation per year/research field (Figures 1, 2).

### 2.2 Co-authorship and collaboration networks

**Figure 3:**
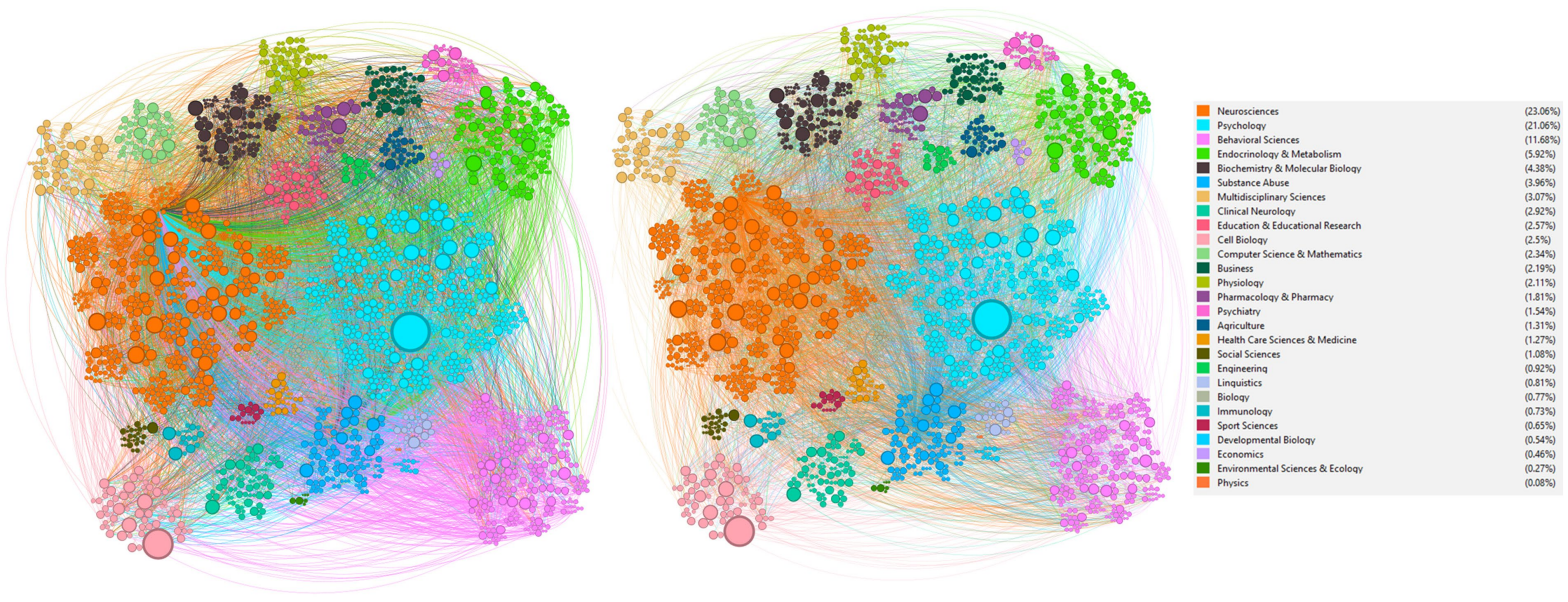
Interdisciplinary cluster network and co-citation analysis: visualizing the structure and citation dynamics in motivated behavior research. Cluster network analysis of research areas and co-citations network, illustrating the interconnectivity and collaborative nature of the field. **(Left)** Edges colored by source citation; **(Right)** Edges colored by target citation. Nodes sized by the global number of citations; edges are based on whithin-network citations. Data span: 1939 to November 2023. Cluster analysis data in text.

We cross reference the doi and title of each paper with the complete reference list of the publications gathered. Each paper (‘node’) received a numeric representation and was linked to the publications that cited it (‘edges’). Each node was colored by its research field (‘clusters’) obtained via Scopus or WoS™ databases. We employed Gephi 0.10 (Bastian et al., 2009) for visual representation (Figure 3); for aesthetic purposes, we applied the Circle Pack Layout hierarchy representation as follows: research field/area, year, and citation count, respectively.

### 2.3 Keyword analysis

Using the ‘Author keywords’ in the dataset, we employed the VoSviewer 1.6.20 (Copyright © 2023 Centre for Science and Technology Studies, Leiden University, The Netherlands) to create a network graph of the 50 most common keywords. Employing similarity measures based on keyword co-occurrence to generate a network visualization, wherein clustering algorithms grouped keywords into clusters according to relatedness, with each cluster differentiated by color. Word cloud code available in the Supplementary material.

### 2.4 Topic modeling

**Figure 4:**
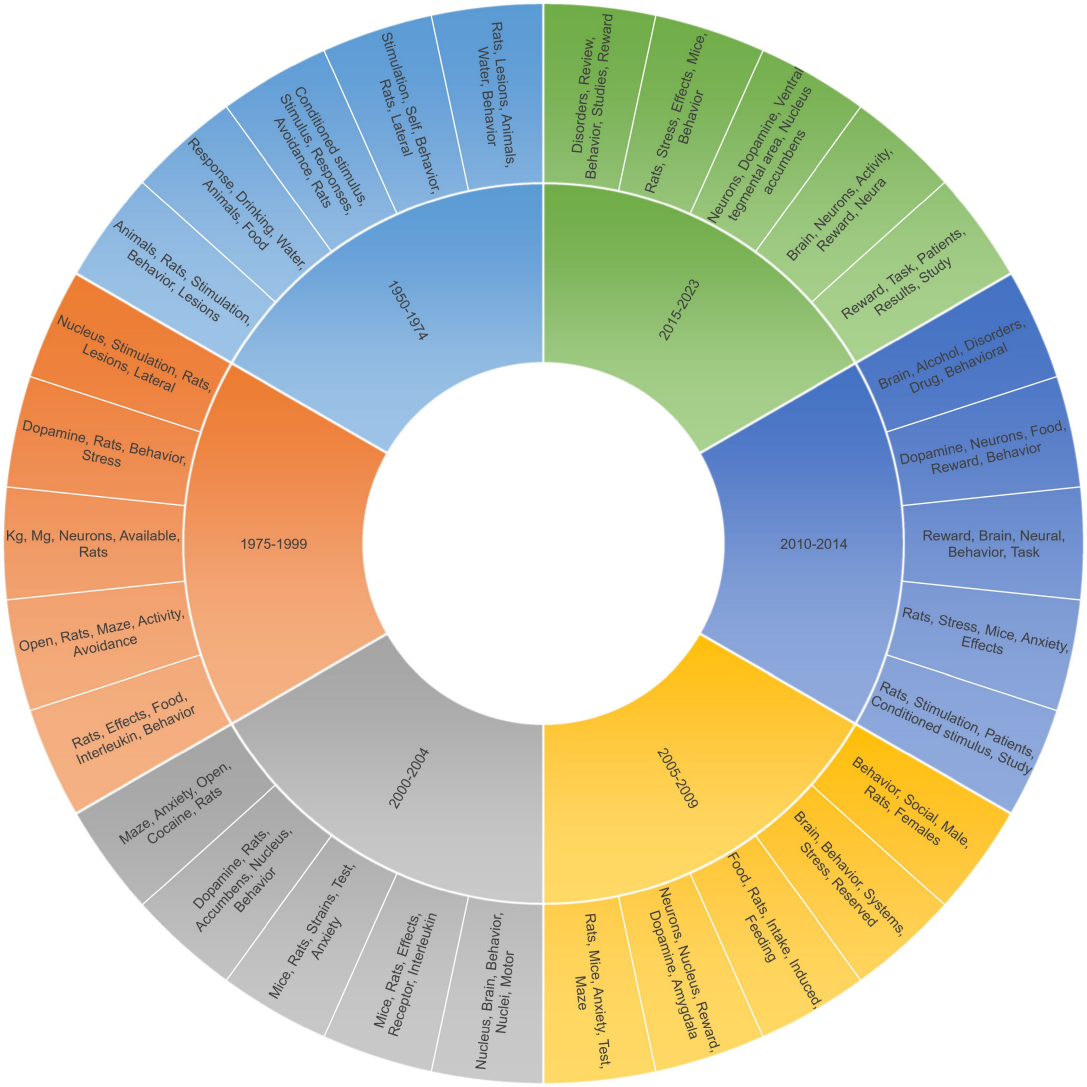
Main keywords in counterclockwise direction per topic in motivated behavior research over time: pie chart containing the 5 most frequent keywords per topic identified by the LDA modeling in motivated behavior research over time. The chart captures the progression of research themes across seven decades, outlined by the predominant topics within each era (further discussion in the text).

To extract meaningful topics from the dataset while minimizing noise and overfitting (Figure 4), providing a comprehensive overview of the thematic landscape within the research corpus on motivated behavior, we employed a Latent Dirichlet Allocation (LDA) with the following parameters:

- Number of Topics (`n_components`): 10
- Maximum Iterations (`max_iter`): 10
- Random State (`random_state`): 0
- Document-Term Matrix Transformation:
  - Vectorization Method: CountVectorizer.
  - `max_df` (Maximum Document Frequency): 0.95
  - `min_df` (Minimum Document Frequency): 2
  - `max_features`: 1000
  - `stop_words`: English

Dissecting the dataset into 10 distinct topics, employing a document-term matrix where terms appearing in more than 95% of the documents are disregarded, as are terms appearing in fewer than 2 documents, with a cap at 1000 features to manage dimensionality and computational efficiency (code available in the Supplementary material).

## 3 Results

The bibliometric analysis yielded some interesting insights into the motived behavior landscape. The compiled dataset allowed us to perform an in-depth analysis of the number of publications and citation frequencies, which revealed important trends and key contributions in the field. Figure 1 illustrates a clear growth in the volume of publications over time, complemented by citation data that underscores the impact of this field. Notably, the graph reflects spiles in average citations that correspond to “groundbreaking” publications that have influenced the subsequent studies. For instance:

- The 1954 peak corresponds to Stellar’s seminal work on the physiology of motivation, which has been cited 373 times, reflecting its foundational influence on subsequent motivational research (Stellar, 1954).
- In the 1980s, we observe heightened citation activity, with Deci and Ryan’s exploration of intrinsic motivational processes and Weiner’s model of motivated behavior collectively receiving over 1,500 citations, indicating a period of significant theoretical advancement in the field (Deci and Ryan, 1980; Weiner, 1980).
- The 1999 peak is attributed to Ikemoto and Panksepp’s integration of nucleus accumbens dopamine function in reward-seeking behavior, which has been cited 1,032 times, highlighting the shift towards a neurobiological understanding of motivation (Ikemoto and Panksepp, 1999).
- Deci and Ryan's (2000) publication, examining the underlying human needs in goal pursuits, stands as the most cited work with an impressive 14,120 citations, underscoring its pivotal role in shaping contemporary motivational theory (Deci and Ryan, 2000).

In addition to the landmark works previously mentioned, the analysis also elucidates two pivotal papers from the early 21st century:

- Kelley and Berridge's (2002) article in the *Journal of Neuroscience* delves into the neuroscience of natural rewards and their relevance to addictive drugs, receiving 1,022 citations. Their work provided a crucial link between the rewarding aspects of natural stimuli and the neurobiological processes that underlie addiction, offering valuable insights into how natural and drug rewards may share common neural pathways (Kelley and Berridge, 2002);
- And a 2012 article published in *Nature*, where Lammel et al. presented a groundbreaking exploration of the ventral tegmental area (VTA), demonstrating input-specific control of reward and aversion. Garnering 946 citations, this study highlighted the nuanced and sophisticated regulation of reward-related behaviors by the VTA, suggesting a more targeted approach to understanding motivational dysfunctions in various psychiatric disorders (Lammel et al., 2012).

These works, particularly those in the last two decades, represent the cutting-edge of motivated behavior research, integrating advanced methodologies and interdisciplinary approaches to address the complex mechanisms of motivation. Kelley and Berridge’s focus on the neural correlates of reward and addiction, along with Lammel et al.’s elucidation of the VTA’s role, represent a shift towards a more intricate understanding of the neurobiological substrates of motivation, further supporting the field’s trajectory towards translational and clinical relevance.

Figure 3 provides a visual representation of the cluster network analysis and co-citations within the field of motivated behavior research. The cluster network analysis denotes a high degree of interdisciplinary collaboration (average degree = 2.565; network diameter = 13; average path length = 3.743), as evidenced by the dense interconnections between diverse research areas such as neuroscience, psychology, and behavioral sciences. This interdisciplinarity is crucial for the advancement of our understanding of motivated behavior, indicating that breakthroughs in the field are the result of increased crosstalk between different scientific domains (Weakly connected components = 685; strongly connected components = 3,131; modularity = 0.566; average clustering coefficient = 0.132). Additionally, the co-citations network further reveals how seminal works serve as nexus points, fostering a dialogue across various disciplines. For instance, the prominence of neuroscience and psychology, as indicated by the larger nodes, features their central role in motivated behavior research.

The network on the left side of the figure illustrates the citation links, with edges colored by source citations, which indicates the foundational research contributing to the area. On the right, the edges are colored by target citations, reflecting the influence of key studies on current research. The size of each node corresponds to the number of global citations, allowing for a quick visual assessment of the most impactful research in the network.

Figure 2 in our review charts the flow of research across various disciplines concerning motivated behavior over an extensive timeline, from 1950 to 2023. This graphical representation elucidates the shifting focus of research areas through the decades, indicating the responsiveness of the academic community to new scientific paradigms and societal needs. The early years show a predominance of studies in neurosciences and psychology, consistent with the foundational efforts to understand the biological underpinnings of behavior. As time progresses, we witness a diversification of research into areas such as endocrinology and metabolism and substance abuse, reflecting an expanding interest in the multifactorial nature of motivation. The turn of the millennium marks a significant increase in publications, particularly within neurosciences, signaling a renaissance of interest possibly spurred by technological advancements in brain imaging and molecular biology techniques. Interestingly, the graph shows a surge in multidisciplinary sciences during this period, emphasizing the trend toward integrative research approaches. In recent years, the data reveals a notable uptick in publications within health care sciences and medicine and social sciences, indicating a broader application of motivated behavior research in addressing complex societal health issues and the interplay of behavioral factors.

Utilizing the LDA model, a popular Natural Language Processing (NLP) technique for topic modeling, we have conducted a detailed analysis of research abstracts within our dataset. This approach has enabled us to identify key topics and observe the evolution of research themes in the study of motivated behavior in the realm of neurosciences. The order does not imply prioritization but rather categorization to facilitate a structured presentation of the findings. Here are the top 10 topics, each with its defining keywords, a brief overview, and specific areas of focus*:

1. Neurochemical Mechanisms: Keywords include receptor, receptors, brain, cholecystokinin (CCK), mice, effect, N-methyl D-Aspartate (NMDA – an excitatory amino acid that works as an agonist of the neurotransmitter glutamate), Gamma-aminobutyric acid (GABA – an inhibitory neurotransmitter), interleukin (IL – proteins that are expressed and secreted by white blood cells, whose role is primarily in the immune system), effects. This topic focuses on the neurochemical processes and interactions in the brain, examining the roles of various receptors and neurotransmitters. It intersects with the “Nucleus Accumbens and Dopamine” theme, which discusses the nucleus accumbens’ role and dopamine’s influence on motivated behavior.^1^
2. Neural Activity and Sleep: Keywords are neurons, dopamine (DA), da, ventral tegmental area (VTA), sleep, cell, neuronal, orexin (a neuropeptide that works as a neuromodulator), mice, and activity. This area delves into the relationship between neural activities and sleep patterns, closely relating to Neuronal Regions and Behavior, which explores interactions in key brain regions like the VTA and cortex.
3. Research Methods and Disorders: Keywords comprise brain, review, neural, disorders, research, behavior, systems, studies, mechanisms, and behavioral. This topic encapsulates methodological approaches and the study of neural disorders, overlapping with Learning and Memory which focuses on brain-based mechanisms.
4. Feeding and Energy Regulation: Keywords include food, behavior, brain, feeding, receptor, intake, receptors, neurons, energy, and effects. It examines neural mechanisms of feeding behaviors and overlaps with Food Intake and Substance Use, discussing food consumption and the effects of substances like cocaine.
5. Circadian Rhythms and Pain: Keywords are circadian, activity, pain, opioid, nucleus accumbens, core part (NACC), rhythms, suprachiasmatic nucleus (SCN), inflammation, depression, and clock. The focus here is on circadian rhythms and pain perception, connected to Stress and Avoidance in Rats which looks at stress responses and conditioning in rats.
6. Addiction and Reward: Keywords consist of nucleus accumbens (NAc), Dopamine (DA), cocaine, drug, induced, rats, accumbens, dopamine receptor I (D1), reward, and stimulation. This topic explores the neural basis of addiction and the reward system, closely related to Dopamine Signaling in Addiction, examining dopamine’s role in addiction and the mesolimbic pathway.
7. Brain Regions: Keywords include nucleus, amygdala, medial, nuclei, lateral, neurons, regions, hypothalamus, hypothalamic, and ventral. It focuses on specific brain regions and their functions, intersecting with Neuronal Regions and Behavior, which explores the interplay between various brain regions and behaviors.
8. Behavioral Studies: Keywords are rats, anxiety, mice, social effects, behavior, stress, test, results, and alcohol. This area investigates behavioral patterns, including Animal Behavior and Sex Differences, examining behavior in animals with a focus on social and sexual contexts.
9. Social and Motor Learning: Keywords comprise basal, ganglia (the correct and official nomenclature is basal nuclei, but the classical use of “ganglia” remains in the literature), motor, striatum, social, cortical, striatal, melanin-concentrating hormone (MCH), learning, and functional. It studies neural underpinnings of social behaviors and motor learning, aligning with “Motivation in Humans,” focusing on human participants’ motivation and social behaviors.
10. Brain Connectivity and Reward: Keywords are reward, cortex, functional, associated brain, regions, activation, connectivity, results, and task. This topic delves into brain connectivity and the reward system, complementing “Reward and Task Performance,” which discusses the brain’s reward system concerning tasks and learning.

Each topic represents a unique facet of motivated behavior research, illustrating the field’s diversity and interconnectivity. From neurochemical mechanisms to social learning and human motivation, these themes collectively offer a comprehensive view of the current state and evolution of research in neuroscience and motivated behavior. Notably, the analysis also underscores the dynamic nature of the field, with shifts in research focus over time aligning with the emergence of new technologies and methodologies. Such shifts are indicative of the field’s responsiveness to new scientific evidence and theoretical developments.

Next, we used the author and index keywords of the bibliography to elaborate a visual chronicle of thematic shifts within the domain of motivated behavior research from 1950 to 2023 (Figure 4). Each segment represents a distinct period, with the associated research themes and main keywords detailed within. The early years are characterized by foundational animal studies, with a particular focus on behavioral responses to *stimuli*. As time progresses, the shift towards neurobiological mechanisms becomes evident, with the 1975–1999 segment highlighting a growing interest in the brain’s structures and the role of neurotransmitters like dopamine. The turn of the millennium sees a broadening of the research scope, delving deeper into comparative studies and the effects of various receptors. From 2005 onwards, a diversification of interests is noted, encompassing social behaviors, reward systems, and the neurobiological underpinnings of stress and addiction. The most recent period, 2015–2023, highlights an increased focus on the neural correlates of reward and the implications of these findings for human behaviors and disorders.

Additionally, the LDA model identified significant shifts in research focus across different periods from 1950 to 2023. This model has helped us categorize topics from the abstracts of papers in our dataset and trace the evolution of research themes over the years.

1950–1974: Early Focuses

- Prominent studies on animal behavior, particularly rats, with emphasis on responses to various stimuli like drinking and lesions.
- Research on self-stimulation behaviors in rats and the effects of lateral brain lesions.
- Exploration of conditioned stimuli (CS), response patterns, and avoidance behaviors in rats.
- Investigations into responses like drinking and feeding behaviors in animals.
- Additional focus on animal studies involving stimulation and the effects of brain lesions.

1975–1999: Shifting Paradigms

- Growing interest in brain structures like the nucleus accumbens, and the effects of stimulation and lesions in rats.
- Increased focus on dopamine (DA) and its impact on rat behavior and stress response.
- Studies involving precise measurements in neuron-related experiments.
- Behavioral experiments using open mazes, analyzing avoidance and activity in rats.
- Explorations of the effects of various stimuli on rat behavior, particularly concerning food intake.

2000–2004: Broadening Research

- Maze-based studies were investigating anxiety, the effects of cocaine, and rat behavior.
- Research on the role of dopamine in rat behavior focuses on areas like the nucleus accumbens.
- Comparative studies between mice and rats, examining behavioral responses in different strains.
- Investigations into the effects of various receptors and interleukins in mice and rats.
- Studies on the role of brain nuclei in motor behavior and general neural activity.

2005–2009: Diverse Interests

- Anxiety and maze tests become a common theme in studies involving rats and mice.
- Increased focus on the role of neurons in reward systems, particularly looking at the amygdala and dopamine.
- Food intake and feeding behaviors in rats, emphasizing on induced behaviors.
- Broad studies on brain-behavior systems, stress responses, and reserved capacities.
- Social behaviors become a focus, especially differences in behavior between male and female rats.

2010–2014: Expanding into Human Contexts

- Utilization of rats in studies relevant to human patients, focusing on conditioned stimuli and broader study implications.
- Research on stress and anxiety effects across rats and mice.
- Explorations into how reward systems in the brain influence behavior and neural processes.
- Studies linking dopamine with food-related behaviors, reward systems, and general behavior.
- A shift towards understanding alcohol-related disorders and drug effects on behavioral patterns.

2015–2023: Current Trends

- A strong focus on reward systems in the context of patient studies and task-related results.
- Investigations into brain neuron activity, particularly relating to rewards and neural processes.
- Detailed studies of dopamine neurons, emphasizing areas like the VTA and NAc.
- Continued research on stress effects in rats and mice, analyzing behavioral patterns.
- Review-based studies on disorders, behavior, and the role of reward systems.

**Figure 5:**
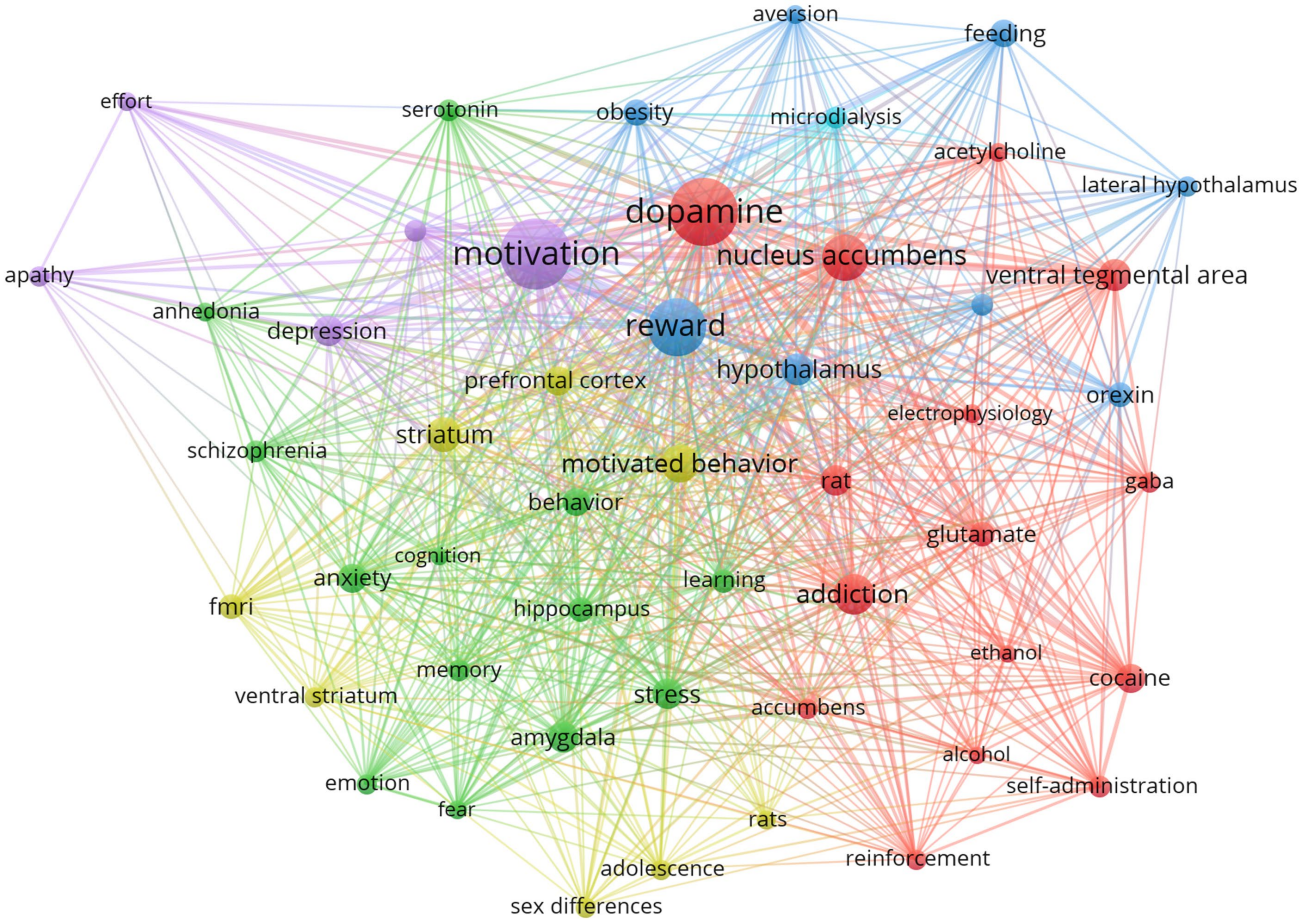
Cluster network of keywords: cluster network of the 50 most used author keywords. This network elucidates the prevalent and significant terms within our dataset. Colored based on co-occurrence (data details in Supplementary material).

The representation in Figure 5 suggests potential functional and conceptual relationships between clustered keywords. The proximity of terms such as ‘reinforcement,’ ‘self-administration,’ ‘addiction,’ ‘glutamate,’ and ‘accumbens’ can be interpreted as reflecting the intense focus on the neural circuitry of addiction. This cluster implies a thematic concentration on the biochemical pathways of reinforcement and the role of specific neurotransmitters and brain regions. Similarly, the adjacency of ‘stress’ and ‘learning’ points to a possible research nexus exploring the effects of stress on cognitive processes. The bibliometric analysis delineates a prominent centrality and interconnectivity of ‘dopamine,’ ‘motivation,’ and ‘reward’ within the *corpus* of neuroscience literature on motivated behavior, as evinced by their substantial link weights and citation frequencies. This data indicates a paradigmatic shift towards more nuanced aspects of motivational processes, particularly focusing on deficits such as ‘anhedonia’ and ‘apathy,’ evidenced by their recent emergence in scholarly discourse. The significant citation metrics for ‘GABA’ and ‘aversion’ emphasize their roles in neuropsychological mechanisms underlying motivated behaviors. The inclusion of terms like ‘FMRI’ reflects the integration of advanced neuroimaging methodologies, underscoring the interdisciplinary nature of the field. The emergence of certain terms with more recent average publication years indicates evolving research trajectories, potentially signaling new functional hypotheses.

**Figure 6:**
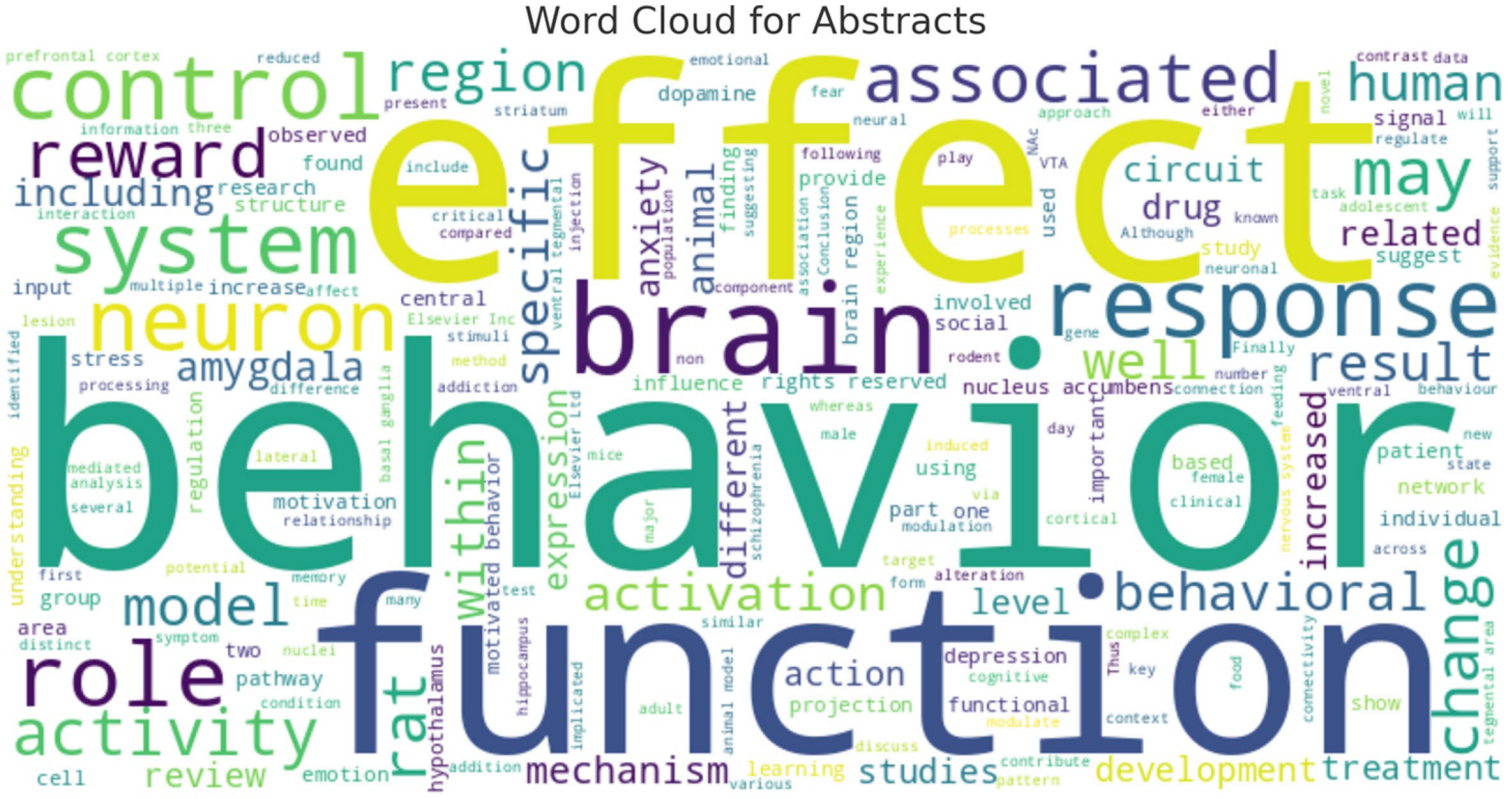
Word cloud of abstract words: word cloud generated from the abstracts of the compiled publications. This visualization emphasizes the frequency of terms, with the size of each word corresponding to its prevalence in the literature. The word cloud provides an at-a-glance understanding of the core topics and terminologies that are prevalent in the study of motivated behavior. Prevalent English words (also known as “stop words”) such as “and” “the” “and of” were removed.

The results presented in Figure 6’s word cloud illustrate once again the multifaceted nature of motivated behavior research. The preeminence of terms such as ‘behavior,’ ‘circuit,’ and ‘neuron’ confirms a strong focus on the neurobiological mechanisms underlying behavioral responses. The collective appearance of terms related to ‘stress,’ ‘addiction,’ and ‘amygdala’ points towards an expanding interest in the pathologies of motivation, which holds relevance for clinical applications. The sizeable representation of ‘human’ in the cloud indicates a significant alignment of the research with human studies, which is critical for the translation of animal model findings into human applications. These findings suggest that the field is not only robust but also dynamic, as evidenced by the varied and evolving focal points over time. The collective appearance of terms related to ‘stress,’ ‘addiction,’ and ‘amygdala’ points towards an expanding interest in the pathologies of motivation, which holds relevance for clinical applications. Overall, the bibliometric analysis reveals a research landscape that is characterized by its depth, with a strong foundation in neuroscience, and its scope, as it integrates findings across multiple domains to enhance our understanding of motivated behavior.

## 4 Discussion

The field of motivated behavior is evidently broad, with research spanning various facets, from memory to learning mechanisms. Our bibliometric analysis has illuminated the expanding interest in the domain, especially in the last two decades. The analytical results, particularly from the cluster networks and word clouds, reinforce the observation that the last two decades have witnessed a burgeoning interest in diverse yet interconnected facets of motivated behavior. From the most cited articles, which serve as foundational pillars, to the diverse themes emerging from the abstracts, we gain a clear understanding of the field’s range. The prominent surge in publications since the early 2000s, as depicted in Figure 1, likely mirrors the technological revolutions in neuroimaging and molecular biology, which have granted us unprecedented access to the workings of the brain. This surge is indicative of a pivotal period where the field began to embrace complex neural mechanisms more fully. The trajectory of research areas over time in Figure 2 echoes this sentiment, revealing a diversification of focus as newer methodologies enable a more nuanced exploration of the brain’s motivational circuitry. Initially, the field was dominated by basic behavioral studies using animal models (Stellar, 1954; Handley and Mithani, 1984), but there has been a gradual transition toward exploring neural mechanisms and human-centric research in recent years (Koenka, 2020). This progression reveals the field’s dynamic nature, evolving from foundational animal studies to advanced explorations in neuroepigenetics, computational modeling, and interdisciplinary collaborations.

The analysis paints a multi-dimensional picture of motivation research, spanning molecular and neurochemical studies to behavioral and cognitive assessments. Each identified topic and associated keywords provide a comprehensive understanding of the prevailing trends and focal points, offering guidance for future research in neuroscience and psychology. As shown in Figure 3, this interdisciplinary dialogue has extended beyond traditional boundaries, incorporating insights from endocrinology, pharmacology, and even more distantly related fields like computer science and mathematics, reflecting a broader recognition of the multifactorial nature of motivation. Notably, recent trends indicate a growing interest in neuromodulation techniques (Kelley and Berridge, 2002), the gut-brain axis’s role in motivation (Morris et al., 2018), and the potential impact of artificial intelligence in understanding and influencing motivated behaviors (Benvenuti et al., 2023). Emerging themes suggest a burgeoning interest in neuroepigenetics, with a focus on how epigenetic modifications influence motivation-related neural circuits (Mews et al., 2021). The integration of computational models with experimental neuroscience is also gaining attraction, promising deeper insights into the neural networks underlying motivated behaviors (Koenka, 2020; Williams, 2023).

In the discussion of recent trends, including the potential for artificial intelligence (AI) in understanding and influencing motivated behavior, we reference emerging studies (e.g., Benvenuti et al., 2023) that illustrate AI’s increasing role in behavioral neuroscience. This highlights the interdisciplinary approaches that extend beyond the conventional scope of our bibliometric analysis. While AI was not a prominent keyword within our dataset, its inclusion in the discussion is predicated on the anticipation of future research trajectories and the increasing integration of computational methods in neuroscience. Similarly, the mention of epigenetics, despite its absence in the keyword clusters of our analysis, is derived from a forward-looking assessment of the field’s evolution. References to neuroepigenetics (e.g., Mews et al., 2021) are included to emphasize the field’s dynamic nature and the emerging interest in how epigenetic mechanisms influence neural circuits driving motivated behaviors.

The collaborative landscape, inferred from the co-authorship networks, highlights a trend towards collective intelligence where the synthesis of expertise across domains is crucial. This tendency towards collaboration is perhaps a natural response to the increasing complexity of the field, necessitating a multidisciplinary approach to unravel the layers of motivated behavior. Another noteworthy aspect of our investigation is the intricate interplay between diverse neural systems in motivated behavior, especially as observed in non-human animal models. While the well-established role of the dopaminergic system in motivation is widely recognized (Kelley and Berridge, 2002; Lammel et al., 2012), our analysis illuminates nuanced insights into the way this system interacts with other brain regions and neurochemical pathways. These studies frequently utilize state-of-the-art techniques, such as optogenetics (Blanco-Centurion et al., 2018) and *in vivo* microdialysis (König et al., 2018), enabling the real-time observation and manipulation of neural activity. Such methodologies have provided us with a more profound understanding of the causal connections between neural activation and motivated behaviors, such as the pursuit of nourishment or drugs. Furthermore, animal studies furnish a controlled setting to investigate the fundamental principles of motivation without the confounding variables that often manifest in human studies. They also present the opportunity to investigate the evolution of these systems across distinct species, thereby offering an evolutionary outlook on motivation. These distinctive aspects are crucial as they contribute to the construction of a bridge from fundamental neuroscience to applied research in human psychology and psychiatry, ultimately serving to inform the development of interventions for disorders related to motivation.

The increase in publications since the 1950s, peaking around 2015, reflects the escalating interest in this domain. Co-authorship networks reveal key authors and their collaboration patterns, accentuating the field’s collective progress. Key research areas such as addiction, reward systems, neural circuitry, sexual behavior, and feeding have been prominently featured, signifying the diverse interests and approaches within the study of motivated behavior. In recent years, new or contemporary theories have emerged, such as an evolved version of Deci and Ryan’s self-determination theory focusing on intrinsic and extrinsic motivation influenced by psychological needs and social factors (Ryan and Deci, 2017; Morris et al., 2022). Other modern theories include organizational justice theory, examining fairness perceptions in reward distribution, and the continued relevance of self-efficacy and reinforcement theories (Berridge, 2018). These recent theories reflect ongoing development in understanding motivation, considering evolving social and organizational contexts.

The interplay between human and animal studies, and the intersection of psychology with animal research, form the cornerstone of our comprehensive understanding of motivated behavior. Animal studies are instrumental in translational research, providing insights into basic neural and behavioral mechanisms that inform human psychology, particularly within areas like neurobiology and behavioral psychology (Rosenblatt, 2003; Pires et al., 2013; Williams, 2023). These studies offer a controlled environment for testing theories, such as operant and classical conditioning, which are foundational to behavioral psychology. Additionally, comparative psychology and ethology contribute to a deeper understanding of behavior in an evolutionary context (Finlay et al., 2011; Filice and Smith, 2023). Studies on motivated behavior in humans and other animals explore neurobiological mechanisms and are framed within similar theoretical constructs like reinforcement and reward. However, they differ significantly in methodology and complexity, with animal studies often employing invasive techniques for detailed brain analysis, which are not feasible in human research. On the other hand, human studies focus on complex social and cognitive aspects of motivation and adhere to strict ethical guidelines. While animal studies lay the groundwork for understanding basic mechanisms, human studies offer insights into more complex and culturally influenced behaviors. This multidisciplinary approach, combining controlled animal experiments with complex human behavior studies, enriches our knowledge across disciplines, leading to advancements in both theoretical understanding and practical applications in human and animal health.

In conclusion, as neuroscience continues to intersect with technological and methodological advancements, our understanding of motivated behavior remains as crucial and relevant as ever. Through our bibliometric lens, we traced the evolution of topics, distinguished its key contributors, and recognized the themes that have developed it. Looking ahead, the convergence of experimental neuroscience, computational models, and clinical insights promises a deeper understanding of motivated behaviors, opening new avenues for addressing neuropsychiatric disorders and enhancing human well-being. This bibliometric analysis offers not just a retrospective glance but also charts a path forward, ensuring that our quest to decipher the brain’s motivation machinery remains unwavering and constantly progressing.

### 4.1 Emerging questions and future directions

1. Neurological underpinnings: How do the neurological mechanisms, especially those associated with dopamine, play into the broader constructs of motivation and behavior?
2. Behavioral manifestations: What are the real-world implications of these findings, especially concerning substance use, learning, and social behaviors?
3. Interdisciplinary collaborations: Given the interconnected nature of the topics (as seen in the co-authorship network), how can further interdisciplinary collaborations augment our understanding of motivated behaviors?
4. Technological advancements: How might future technological advancements reshape research in this domain?

### 4.2 Limitations

Our bibliometric review, which focuses on analyzing and visualizing large datasets from academic publications, stands out for its ability to map trends, identify key contributors, and highlight recurrent research topics. Unlike narrative or systematic reviews, bibliometric analysis provides a quantitative outlook, offering insights into publication patterns, citation networks, and emerging areas within a field. This approach is particularly useful in fields like neuroscience, where understanding the evolution of research themes and the interplay between different sub-domains is crucial. Nonetheless, bibliometric reviews have their limitations. They rely heavily on available data and citation metrics, which may not fully capture the qualitative aspects of research impact or the intricacies of theoretical developments (Romanelli et al., 2021). One inherent limitation of this method is the potential for incomplete data compilation during the data mining process. Various factors might result in some relevant publications not being included in the final dataset analyzed, thereby impacting the comprehensiveness of the review (for instance, Maslow’s theory of human motivation in our final dataset) (Maslow, 1943). We recognize that the selected bibliometric analysis method led to the exclusion of this paper. However, a different approach that considers the analysis of cited references would have included this author. Regarding Maslow’s theory from 1943, it is crucial to acknowledge its historical significance while also recognizing the controversies and limitations it presents. Maslow’s hierarchy of needs has been a foundational model in understanding human motivation, yet it faces criticism for its hierarchical structure and the assumption that lower needs must be satisfied before higher needs can be pursued. This discussion serves as an example of how a historical review can provide context to theories, a task that is beyond the scope of a bibliometric review. This is where other review types, like critical reviews or qualitative systematic reviews, complement bibliometric analysis (Grant and Booth, 2009). These reviews delve deeper into the content and quality of research, providing nuanced interpretations and theoretical insights that are not immediately apparent in quantitative data. While a bibliometric review offers quantitative insights into research trends and patterns, a historical review provides qualitative context and theoretical evolution. Both approaches, despite their distinct methodologies, can complement each other, offering a more comprehensive understanding of the field.

The choice of input search terms indeed forms a critical aspect of our bibliometric analysis and subsequent inferences. While our search term focused primarily on “motivated behavior,” it’s important to acknowledge that this selection strategy inherently shapes the scope and scale of the literature included in our review. We agree that articles incorporating the term “motivation” may represent relevant contributions to the broader field of motivated behavior research. In retrospect, a broader set of search terms, including variations such as “motivation,” “reward-seeking behavior,” “incentive motivation,” and related terms, could have potentially captured a more comprehensive range of relevant literature. Our decision to focus specifically on “motivated behavior” stemmed from our aim to provide a focused examination of the behavioral aspects of motivation and their neural underpinnings. However, we acknowledge that this approach may have inadvertently excluded some relevant studies that use alternative terminology or conceptual frameworks. The choice of “Motivated Behavior” as our search term was deliberate. We sought to focus our analysis on literature explicitly examining the construct of motivated behavior, rather than the broader concept of “motivation.” This precision was necessary to maintain computational feasibility and relevance to our study’s scope. In our revised methodology section, we now clarify the rationale behind our search term selection and its implications for the research findings. For example, many publications use “motivation” as a “noun” without specifying a cause or an aim for a certain behavior. When looking for documents in Scopus with the query “Motivation,” over 1,000,000 publications show up, making the bibliometric analysis used almost unfeasible due to computing limits.

The relatively low number of publications in clinical neurology compared to topics like substance abuse and addiction may stem from the way these databases categorize research articles. Articles on clinical neurology that discuss motivated behaviors such as apathy may be classified under broader categories or specific subfields, which could result in their underrepresentation in a category labeled “clinical neurology.” The prominence of substance abuse and addiction in the dataset likely reflects the high volume of research output in these areas that explicitly mention “motivated behavior,” which is a core element in the study of addiction. The neurobiological mechanisms underlying addiction are often framed explicitly within the context of motivated behaviors, making them more likely to be captured by our search criteria.

Our analysis does not currently distinguish between articles based on animal or human behavior, which is a valid point of curiosity. While the document offers a general analysis, specifying the proportion of studies focused on each could provide a more detailed understanding of the field’s research directions. Moreover, incorporating this distinction into the analysis and discussions could shed light on how different types of motivated behavior – such as survival instincts, social needs, or well-being – may be represented in the literature. This additional layer of analysis could explain certain bibliometric trends and could be particularly relevant in understanding the nuances behind the publication plateau observed in recent years.

## Author contributions

AH: Formal analysis, Investigation, Methodology, Software, Writing – original draft. JB: Conceptualisation, Funding acquisition, Project administration, Resources, Supervision, Validation, Visualization, Writing – original draft, Writing – review & editing.

## Footnotes

^1^*The naming of each topic was derived directly from the aggregation of keywords that frequently co-occur within the research abstracts. These terms were selected based on their statistical significance and relevance to the respective thematic content they represent. For instance, “Neurochemical Mechanisms” accurately denotes the topic’s focus on the biochemical processes within the brain, while “Neural Activity and Sleep” encapsulates the research concerning neural functions in relation to sleep patterns.
